# Association Between Periodontitis and Nosocomial Pneumonia: A Systematic Review and Meta-analysis of Observational Studies 

**DOI:** 10.3290/j.ohpd.a44114

**Published:** 2020-04-01

**Authors:** Laura Silva Jerônimo, Lucas Guimarães Abreu, Fabiano Araújo Cunha, Rafael Paschoal Esteves Lima

**Affiliations:** a Graduate Student, Department of Clinical, Pathology and Dental Surgery, Federal University of Minas Gerais, Belo Horizonte, MG, Brazil. Idea, methodological design, definition of search strategy, search and selection of articles, data extraction and qualitative analysis, wrote the manuscript.; b Professor, Department of Child and Adolescent Oral Health, Federal University of Minas Gerais, Belo Horizonte, MG, Brazil. Methodological design, definition of search strategy, synthesis of results, wrote the manuscript.; c Professor, Department of Clinical, Pathology and Dental Surgery, Federal University of Minas Gerais, Belo Horizonte, MG, Brazil. Methodological design, definition of search strategy, wrote the manuscript.; d Professor, Department of Clinical, Pathology and Dental Surgery, Federal University of Minas Gerais, Belo Horizonte, MG, Brazil. Idea, methodological design, definition of search strategy, search and selection of articles, data extraction and qualitative analysis, wrote the manuscript.

**Keywords:** meta-analysis, hospitalisation, inpatient care units, periodontitis, pneumonia, ventilator associated

## Abstract

**Purpose:**

To assess the relationship between periodontitis and nosocomial pneumonia in intensive care unit (ICU) patients.

**Materials and Methods:**

The present study was conducted in accordance with the guidelines of the Preferred Reporting Items for Systematic Reviews and Meta-Analyses (PRISMA) statement and registered (CRD42018105124) with PROSPERO (International prospective register for systematic reviews, University of York, York, UK). A search was conducted in five databases without restrictions regarding language or date of publication. From 560 studies selected, 10 underwent full-text analysis. Five studies were eligible (five case-control studies), and all were entered in the meta-analysis. Meta-analysis was performed with tests for sensitivity and statistical heterogeneity. Summary effect measures were calculated by odds ratio (OR) and 95% confidence interval (CI).

**Results:**

There was a significant association between periodontitis and nosocomial pneumonia in the meta-analysis (OR 2.55, 95% CI 1.68 to 3.86). In this meta-analysis, I^2^ = 0%.

**Conclusions:**

The evidence demonstrates a positive association between periodontitis and nosocomial pneumonia. Individuals with periodontitis admitted to the ICU were more likely to present nosocomial pneumonia than individuals without periodontitis.

The aetiology of periodontitis, an inflammatory condition of dental support tissues, is associated with bacteria. It is characterised by changes in clinical parameters, such as probing depth, attachment loss, and bleeding on probing. Tooth mobility, halitosis, and gingival recession also occur.^18^ The inflammatory process initiated in response to bacteria aggression is the main cause of the destruction of periodontal structures observed in periodontitis.^1^ Periodontitis has been associated with many systemic conditions, such as diabetes, cardiovascular diseases,^7^ atherosclerosis^16^ and respiratory diseases, e.g. pneumonia.^15^

Nosocomial pneumonia is an infection of the lungs usually caused by bacteria,^5^ which the patient may acquire at least 48-72 h after admission to the hospital. Nosocomial pneumonia has been recognised as an important public health issue worldwide, due to its association with high rates of morbidity and mortality among hospitalised individuals. High costs are also incurred for affected individuals and the healthcare system, as the therapeutic demand and the length of hospital stay significantly increase.^20^ Ventilator-associated pneumonia is the most common nosocomial infection in intensive care units (ICU). Ventilator-associated pneumonia may take place after mechanical ventilation (endotracheal tube or tracheostomy) is provided. The incidence varies between 9% and 40%.^24^

In individuals under intensive care, oral hygiene may be an issue. Bacterial colonisation of dental biofilm and periodontal disease may have an important role as reservoirs of microorganisms that cause nosocomial pneumonia, since the latter results from aspiration of pathogens from the oral cavity and oropharynx into the lower respiratory tract. The dental biofilm of patients admitted to intensive care units may be colonised by potential respiratory pathogens. In this regard, periodontitis may be a contributing factor for the development of nosocomial pneumonia.^8^ The accumulation of oral pathogens changes the environmental conditions of the mouth, leading to airway infection by new pathogens.^22^ The aspiration of small amounts of secretion from the oral cavity is common in healthy individuals, in particular during sleep. Among patients with altered levels of consciousness, however, the amount of aspirated secretion tend to increase.^3^

The objective of this systematic review and meta-analysis was to evaluate the association between periodontitis and nosocomial pneumonia among individuals admitted to the intensive care unit. The present research question was whether individuals admitted to the ICU with periodontitis are more likely to develop nosocomial pneumonia than individuals admitted to the ICU without periodontitis.

The following PECO question was applied: ‘Are individuals with periodontitis who are admitted to the ICU more likely to develop nosocomial pneumonia than individuals without periodontitis?’

- P (Patients) = individuals admitted to the ICU
- E (Exposure) = periodontitis
- C (Comparison) = no periodontitis
- O (Outcome) = nosocomial pneumonia

## Materials and Methods

### Protocol and Registration

This systematic review and meta-analysis was conducted in accordance with the guidelines of the Preferring Reporting Items for Systematic Reviews and Meta-analyses (PRISMA).^19^ The protocol was registered in the International Prospective Register of Systematic Reviews (PROSPERO) under the registration number CRD42018105124.

### Eligibility Criteria

Studies were included which compared individuals with periodontitis and individuals without periodontitis who were admitted to the ICU regarding the occurrence of nosocomial pneumonia. Cross-sectional, case-control, or clinical-trial studies that presented data of interest for the present systematic review and meta-analysis were eligible for inclusion. Literature reviews, studies published in meeting abstracts, and studies with no data on the periodontal condition of participants were excluded.

### Information Sources

The search was conducted by two of the present authors (LSJ and RPEL) in April, 2018. The following electronic databases were used: Pubmed, Web of Science, Scopus, Medline Ovid, and Lilacs. Restrictions on study language or date of publication were not imposed.

### Search Strategy

The following search strategy was used in the electronic databases: ((periodontitis OR periodontal pocket OR adult periodontitis OR prepubertal periodontitis OR juvenile periodontitis OR periodontal disease OR chronic periodontitis OR aggressive periodontitis) AND (pneumonia OR pneumonia nosocomial OR pneumonia, ventilator-associated OR ventilator-induced lung injury)).

In the case of missing data or when the article had not been published yet, the authors were contacted to retrieve additional information or the article in full.

Endnote Web software (Clarivate Analytics; Toronto, Canada) was used to organise the studies.

### Selection of Studies

References retrieved through the electronic search were screened using the eligibility criteria. Two independent authors (LSJ and RPEL) screened the references. Initially, titles/abstracts were evaluated. If the title/abstract did not contain sufficient information, the respective full text was also evaluated. The studies that fulfilled the eligibility criteria were included in this systematic review and meta-analysis. Disagreement between the two review authors on study selection was resolved by means of discussion and consensus.

### Data Extraction Process and Extracted Items

The following data were extracted from each included study: author name(s) and year of publication, country in which the study was conducted, sample size, participants’ age, diagnostic criteria for periodontitis, diagnostic criteria for nosocomial pneumonia. and main results.

### Methodological Quality of the Studies

The methodological quality of the included studies was evaluated by means of the Newcastle-Ottawa scale.^27^ This scale has eight items distributed across three categories: 1. selection of the study groups (adequate definition of cases, representativeness of the cases, selection of controls and definition of controls); 2. comparability between groups (adjustment for confounders); and 3. evaluation of the exposure/outcome of interest (ascertainment of exposure, same method of ascertainment for cases and controls, and nonresponse rate). Each item in the ‘selection of the study groups’ and ‘evaluation of the exposure/outcome of interest categories could be awarded 1 point. The item ‘comparability between groups’ could be awarded 2 points. Therefore, the score ranges from 0 to 9 points. The methodological quality of the included studies was evaluated independently by two authors (LSJ and RPEL). Disagreements were resolved by consensus.

### Synthesis of Results

The articles with methodological homogeneity were incorporated into the meta-analysis. Statistical heterogeneity was evaluated using I^2^statistics.^17^

## Results

### Selection of Studies

**Fig 1:**
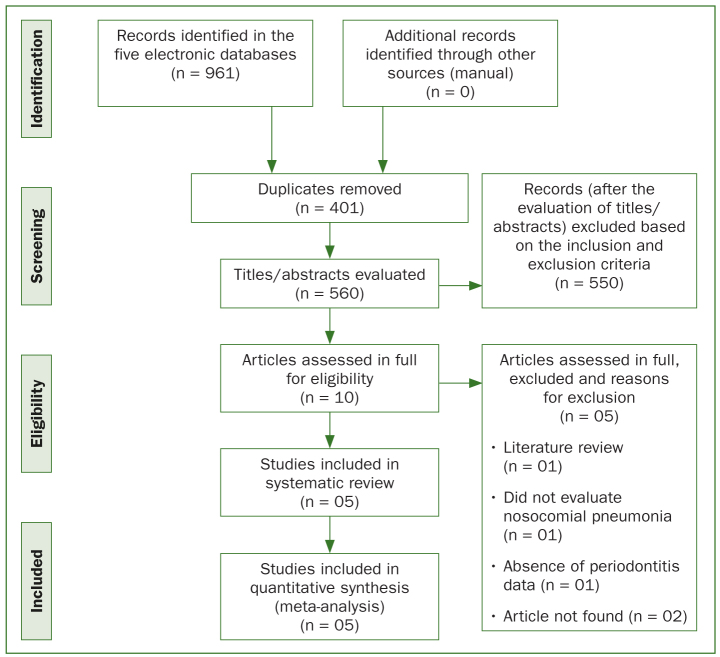
Flowchart of the systematic review, describing the search and the selection of the included articles.

A total of 961 records were identified in the 5 electronic databases, of which 401 were duplicates and were excluded. Among the 560 studies retrieved in the electronic search, 10 studies^2,4,6,10,11,12,14,15,22,26^ were selected for full-text assessment. After the evaluation of the full texts, 5 studies^2,10,14,15,22^ were included in this systematic review and meta-analysis (Fig 1).

### Characteristics of the Studies

The main characteristics of the included studies are displayed in Table 1. Four studies^2,10,14,15^ were published in English and one^22^ in Portuguese. The sample of the 5 studies consisted of a group of individuals with nosocomial pneumonia and a control group with individuals without nosocomial pneumonia. The number of participants in each study ranged from 23 to 315 individuals. A total of 163 individuals with nosocomial pneumonia and 371 individuals without nosocomial pneumonia were evaluated. In the 5 articles,^2,10,14,15,22^ the diagnostic criteria for periodontitis were provide, but only four of them^2,14,15,22^ described the diagnostic criteria for nosocomial pneumonia for sample selection.

**Table 1:** Characteristics of the included studies

Authors	Study design	Country	Sample (n)	Age (years)	Duration of hospitalisation (days)	Diagnostic criteria for pediodontal disease	Diagnostic criteria for nosocomial pneumomia	Outcome
Almondes et al (2017)			NP: 20 Control: 40	> 40 years NP: 12 (60.0%) Control: 21 (52.5%) ≤ 40 years NP^a^: 8 (40.0%) Control: 19 (47.5%) p = 0.581	≥ 5 days NP: 13 (65.0%) Control: 13 (32.5%) < 5 days NP: 7 (35.0%) Control: 27 (67.5%) p = 0.016	≥ 4 teeth with 1 ≥ sites with PD ≥ 4 mm, CAL ≥ 3 mm and BOP at the same site	Diagnosis performed by the medical team of the institution’s ICU and based on the following criteria: presence of recent infiltrate identified in chest radiograph associated with fever, leukocytosis or leukopenia; cough or purulent sputum; bacterial growth in tracheal aspirate culture, present after at least 48 h of hospitalisation.	NP: 05 (25.0%) Control: 05 (12.5%) p = 0.220; OR 2.33 (0.58 -9.26)
De Marco et al (2013)	Case-control	Brazil	NP: 7 Control: 16	NP: 56.28 ± 10.85 years Control: 51.62 ± 14.0 years p ≤ 0.46	NP: 13.57 ± 7.24 days Control: 10.6 ± 6.89 days p < 0.39	Periodontal disease index	Not reported	NP: 06 (85.7 %) Control: 12 (75.0%) p = <0.9
Pinheiro et al (2007)	Case-control	Brazil	NP: 29 Control: 4	Not reported	Not reported	Score 3 or 4 of the CPI in at least one sextant	Radiographically presents at least one of the characteristics of persistent infiltrate, consolidation or cavitation and, clinically, at least one of these characteristics: fever, leukocytosis (> 12,000 leukocytes /mm^3^), leukopenia (<4000 leukocytes /mm^3^), or moderate to severe secretion.	NPa: 22 (75.9%) Control: 03 (75.0%) p = 1.000
Gomes-Filho et al (2009)	Case-control	Brazil	NP: 22 Control: 81	≤ 35 years NP: 14 (63.6%) Control: 44 (54.3%) >35 years NP: 8 (36.4%) Control: 37 (45.7%) p = 0.44	≤ 5 days NP: 12 (54.5%) Control: 60 (74.1%) > 5 days NP: 10 (45.5%) Control: 21 (25.9%) p = 0.07	≥ 4 teeth with ≥ 1 sites with PD ≥ 4 mm, CAL ≥ 3 mm and BOP at the same site	If, within 48h of initial hospitalisation, clinical examination revealed the presence of a dull sound on percussion or crackling rales or chest radiographic evidence of new or progressive inﬁltration, consolidation, cavitation or pleural effusion. In addition, one of the following was required to make a diagnosis: appearance of purulent sputum or changes in the sputum characteristics that existed at the time of hospitalisation; microorganisms *(Pseudomonas aeruginosa, Pseudomonas sp., Klebsiella sp., E. coli, Acinetobacter baumannii, Staphylococcus aureus, Streptococcus pneumoniae, Citobacter freundi, Klebsielle pneumoniae* and* Citobacter amalonaticus)* isolated from blood culture, isolated from bronchoalveolar lavage; and histological evidence of pneumonia from bronchial lavage.	NP: 11 (50%) Control: 30 (37%) p = 0.27; OR 1.70 (0.60-4.87)
Gomes-Filho et al (2014)	Case-control	Brazil	NP: 85 Control: 230	18 to 42 NP: 35 (41.18%) Control: 132 (57.39%) >42 NP: 50 (58.82%) Control: 98 (42.61) p = 0.01	≤ 5 days NP: 06 (7.1%) Control: 118 (51.3%) > 5 days NP: 79 (92.9%) 112 (48.7%) p = 0.00	≥ 4 teeth with 1 ≥ sites with PD ≥ 4 mm, CAL ≥ 3 mm and BOP^c^	1) Underlying ﬂuid density or dullness on percussion, crackles on clinical examination of the chest, and one of the following: appearance of purulent sputum or change in existing features of the sputum at hospital admission; microorganisms isolated from blood cultures; microorganism isolated in bronchoalveolar lavage or lung biopsy; or histologic evidence of pneumonia; or 2) chest radiograph shows a new or progressive inﬁltration, consolidation, cavitation, or pleural effusion, together with any of the signs mentioned above.	NP: 56 (65.9%) Control: 89 (38.7%) p = 0.00; OR 3.06 (1.82-5.15)

NP = nosocomial pneumonia; PD = probing depth; BOP = bleeding on probing; CPI = community periodontal index; CAL = clinical attachment level; OR = odds ratio.

### Results of the Individual Studies

The prevalence of periodontitis ranged from 25.0% to 85.7% in the groups of individuals with nosocomial pneumonia and from 12.5% to 75.0% in the groups of individuals without nosocomial pneumonia. One study^14^ showed a significant difference between individuals with nosocomial pneumonia and individuals without nosocomial pneumonia in relation to the prevalence of periodontitis.

Of the studies included in this systematic review and meta-analysis, two studies^2,10^ presented data on the plaque index. No difference between individuals with and without nosocomial pneumonia was observed.

### Evaluation of the Methodological Quality of Included Studies

The evaluation of the methodological quality of the included studies is shown in Table 2. The studies received between 4 and 7 of the 9 points possible. Two studies^14,15^ received 7 points, one study^2^ received 6 points, one study^22^ received 5 points, and one study^10^ received 4 points. The main deficiencies identified in the studies were selection of controls and the lack of information on nonresponse rate.

**Table 2:** Study qualification according to Newcastle-Otawa Scale (NOS)

Studies		Almondes et al (2017)	Pinheiro et al (2007)	Gomes-Filho et al (2009)	Gomes-Filho et al (2014)	De Marco et al (2013)
Study design		Case-control	Case-control	Case-control	Case-control	Case-control
Selection: just one star (*) given for each question	1) Is the case definition adequate? a) yes, with independent validation* b) yes, record linkage or based on self-reports c) no description	a*	a*	a*	a*	c
	2) Representativeness of the cases a) consecutive or obviously representative series of cases* b) potential for selection biases or not stated	a*	a*	a*	a*	a*
	3) Selection of controls a) community controls* b) hospital controls c) no description	b	b	b	b	b
	4) Definition of controls a) no history of disease (endpoint)* b) no description of source	a*	a*	a*	a*	a*
Comparability: to 2 stars (*) given for each question	1) Comparability of cases and controls on the basis of the design or analysis a) study controls for age* b) study controls for duration of hospitalisation*	*	-	**	**	-
Exposure: up to 1 star (*) given for each question	1) Ascertainment of exposure a) secure record (e.g. surgical records)* b) structured interview blinded to case/control status* c) interview not blinded to case/control status d) written self-report or medical record only e) no description	a*	a*	a*	a*	a*
	2) Same method of ascertainment for cases and controls a) yes* b) no	a*	a*	a*	a*	a*
	3) Nonresponse rate a) same rate for both groups* b) nonrespondents described c) rate different and no designation	b	c	b	c	c
Maximum number of stars		6	5	7	7	4

### Synthesis of Results (Meta-analysis of the studies)

**Fig 2:**
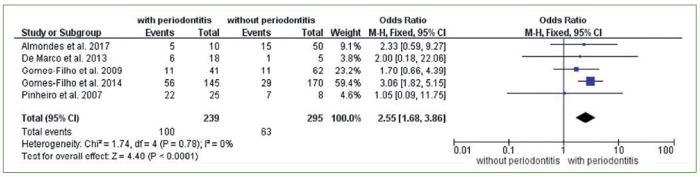
Meta-analysis of five studies evaluating the association between periodontitis and nosocomial pneumonia.

Five articles were included in the meta-analysis.^2,10,14,15,22^ Individuals with periodontitis were 2.55 times more likely to present nosocomial pneumonia than individuals without periodontitis (OR = 2.55; CI = 1.68–3.86). The meta-analysis presented statistical heterogeneity equal to 0% (I^2^ = 0%). Therefore, the fixed model was used (Fig 2).

## Discussion

Due to the significant prevalence and the burden caused by nosocomial pneumonia, it is important to identify factors that may be associated with this disease. This systematic review and meta-analysis suggests that individuals with periodontitis were more likely to present nosocomial pneumonia than their peers without periodontitis. The association between these two diseases is indeed biologically realistic, because the proliferation of bacteria in individuals with periodontitis may lead to the colonisation of the oropharynx, which, ultimately, favors the direct aspiration of pathogens and perpetuates the infection by means of inflammatory and immunologic intermediary factors.^14^ In this sense, periodontitis may contribute to the accumulation of microorganisms in the lung parenchyma and alteration of the respiratory tract epithelium, contributing to the development of nosocomial pneumonia.^25^

Age has been considered a risk factor for nosocomial pneumonia.^9^ Elderly individuals present alterations in the mucosa, increasing their susceptibility to oropharyngeal colonisation and diminishing their ability to defend against diseases.^14^ Among the five studies included in this systematic review and meta-analysis, one^22^ did not present data regarding age. In three studies,^2,10,15^ individuals with and without pneumonia were matched with respect to age. However, in the other study,^14^ there was a significant difference between groups regarding age. Individuals with nosocomial pneumonia were older compared to individuals without nosocomial pneumonia. In addition, the duration of hospitalisation is another factor that should be considered. Longer hospital stays decrease salivary secretion and promote changes in the microbial oral flora in a matter of a few weeks. These conditions favor the prevalence of Gram-negative bacteria and, consequently, make pulmonary infections possible by aspiration of these pathogens.^13,23^ Among the five studies included in this systematic review and meta-analysis, one did not present data regarding duration of hospitalisation.^22^ In other studies,^10,15^ individuals with and without pneumonia were matched with respect to duration of hospitalisation. However, in some studies,^2,14^ there was a significant difference between groups regarding duration of hospitalisation. Individuals with nosocomial pneumonia are hospitalised longer compared to individuals without nosocomial pneumonia.

In studies assessing periodontal outcomes, accuracy in the diagnosis of periodontitis is mandatory. The five studies included in this systematic review and meta-analysis presented different diagnostic criteria for periodontitis. In three of them,^2,14,15^ the diagnosis of periodontitis was based on clinical periodontal parameters, such as probing depth, attachment loss, and bleeding on probing, while in another study,^22^ the criteria were based on the Community Periodontal Index (CPI) for diagnosis of periodontitis. In the remaingin study,^10^ the criteria were based on the Periodontal Disease Index. The diagnosis of periodontitis should be made using periodontal clinical parameters. The use of CPI or Periodontal Disease Index may be biased. It is important to note that the criteria used for the diagnosis of periodontitis have a great impact on the reported prevalence of the disease. Periodontal diseases differing in terms of extension and severity may present different systemic behaviors. Moreover, differences in the diagnostic criteria of nosocomial pneumonia were observed among the included studies. Characteristics of radiographic examination, such as infiltration, consolidation, or cavitation were observed in three studies.^14,15,22^ Clinical characteristics such as fever, leukocytosis, and leukopenia were observed in two studies.^2,14^

The results of this systematic review and meta-analysis underscore the role of oral health providers in ICUs. The information presented here may be helpful to physicians and nurses regarding the effects of poor oral health on the likelihood of the occurrence of nosocomial pneumonia. Hospitalised individuals should be submitted to oral examination for assessment of their oral condition. Those with poor oral health should be given oral health care and monitored.^21^

This systematic review and meta-analysis has certain shortcomings. The quality assessment demonstrated that five of the included studies evaluating the association between periodontitis and nosocomial pneumonia had methodological limitations. However, our study is strengthened by the low statistical heterogeneity of the meta-analysis. Future research in different populations and with larger samples should be conducted to consolidate the knowledge on the association between these two diseases. More controlled studies examining similarities between individuals with and without nosocomial pneumonia are encouraged. It is important that in subsequent studies the potential confounding factors in the possible association between periodontal disease and nosocomial pneumonia be carefully controlled, with no disparities between cases and controls. Microbiological studies evaluating samples of respiratory pathogens associated with the disease should also be carried out. It is also important to conduct intervention studies to evaluating the impact of periodontal treatment on the incidence of nosocomial pneumonia in ICUs.

## Conclusion

Within the limitations of the included studies, our systematic review and meta-analysis of observational data suggests an association between periodontitis and nosocomial pneumonia in ICU patients. Accordingly, ICU patients with periodontitis might be more likely to develop nosocomial pneumonia than those without periodontitis. However, the data is insufficient to draw firm conclusions.
